# Evaluation of Hepatitis A Vaccine in Post-Exposure Prophylaxis, The Netherlands, 2004-2012

**DOI:** 10.1371/journal.pone.0078914

**Published:** 2013-10-17

**Authors:** Jane Whelan, Gerard J. Sonder, Lian Bovée, Arjen Speksnijder, Anneke van den Hoek

**Affiliations:** 1 Department of Infectious Diseases, Amsterdam Public Health Service (GGD), Amsterdam, The Netherlands; 2 Department of Internal Medicine, Division of Infectious Diseases, Tropical Medicine and AIDS, Academic Medical Center, Amsterdam, The Netherlands; 3 Laboratory of Public Health, Amsterdam Public Health Service (GGD) Amsterdam, The Netherlands; Institut Pasteur of Shanghai, Chinese Academy of Sciences, China

## Abstract

**Background:**

The secondary attack rate of hepatitis A virus (HAV) among contacts of cases is up to 50%. Historically, contacts were offered immunoglobulin (IG, a human derived blood product) as post-exposure prophylaxis (PEP). Amid safety concerns about IG, HAV vaccine is increasingly recommended instead. Public health authorities’ recommendations differ, particularly for healthy contacts ≥40 years old, where vaccine efficacy data is limited. We evaluated routine use of HAV vaccine as an alternative to immunoglobulin in PEP, in those considered at low risk of severe infection in the Netherlands.

**Methods:**

Household contacts of acute HAV cases notified in Amsterdam (2004-2012) were invited ≤14 days post-exposure, for baseline anti-HAV testing and PEP according to national guidelines: immunoglobulin if at risk of severe infection, or hepatitis A vaccine if healthy and at low risk (aged <30, or, 30-50 years and vaccinated <8 days post-exposure). Incidence of laboratory confirmed secondary infection in susceptible contacts was assessed 4-8 weeks post-exposure. In a vaccinated subgroup, relative risk (RR) of secondary infection with estimated using Poisson regression.

**Results:**

Of 547 contacts identified, 191 were susceptible to HAV. Per-protocol, 167 (87%) were vaccinated (mean:6.7 days post-exposure, standard deviation(sd)=3.3) and 24 (13%) were given immunoglobulin (mean:9.7 days post-exposure, sd=2.8). At follow-up testing, 8/112 (7%) had a laboratory confirmed infection of whom 7 were symptomatic. All secondary infections occurred in vaccinated contacts, and half were >40 years of age. In healthy contacts vaccinated per-protocol ≤8 days post-exposure, RR_ref. ≤15 years_ of secondary infection in those >40 years was 12.0 (95%CI:1.3-106.7).

**Conclusions:**

Timely administration of HAV vaccine in PEP was feasible and the secondary attack rate was low in those <40 years. Internationally, upper age-limits for post-exposure vaccination vary. Pending larger studies, immunoglobulin should be considered PEP of choice in people >40 years of age and those vulnerable to severe disease.

## Introduction

Hepatitis A virus (HAV) causes acute inflammation of the liver and is transmitted via the fecal-oral route. The virus is endemic in developing countries where sanitary conditions are suboptimal and ≥90% of infections occur in children, are asymptomatic, and lead to lifelong immunity [1]. Incidence of symptomatic infection increases with age and 80% of adults infected will complain of symptoms [2]. In industrialised, low-endemic regions such as the Netherlands, population seroprevalence of antibodies is low and much of the adult population is susceptible if exposed. Spread is typically limited to travelers to HAV endemic countries, men who have sex with men (MSM) and contacts of hepatitis A patients. The secondary attack rate can be up to 50%, and though the infection is often mild and self-limiting, HAV infection can in rare cases, lead to fulminant hepatic failure [3]. 

**Figure 1:**
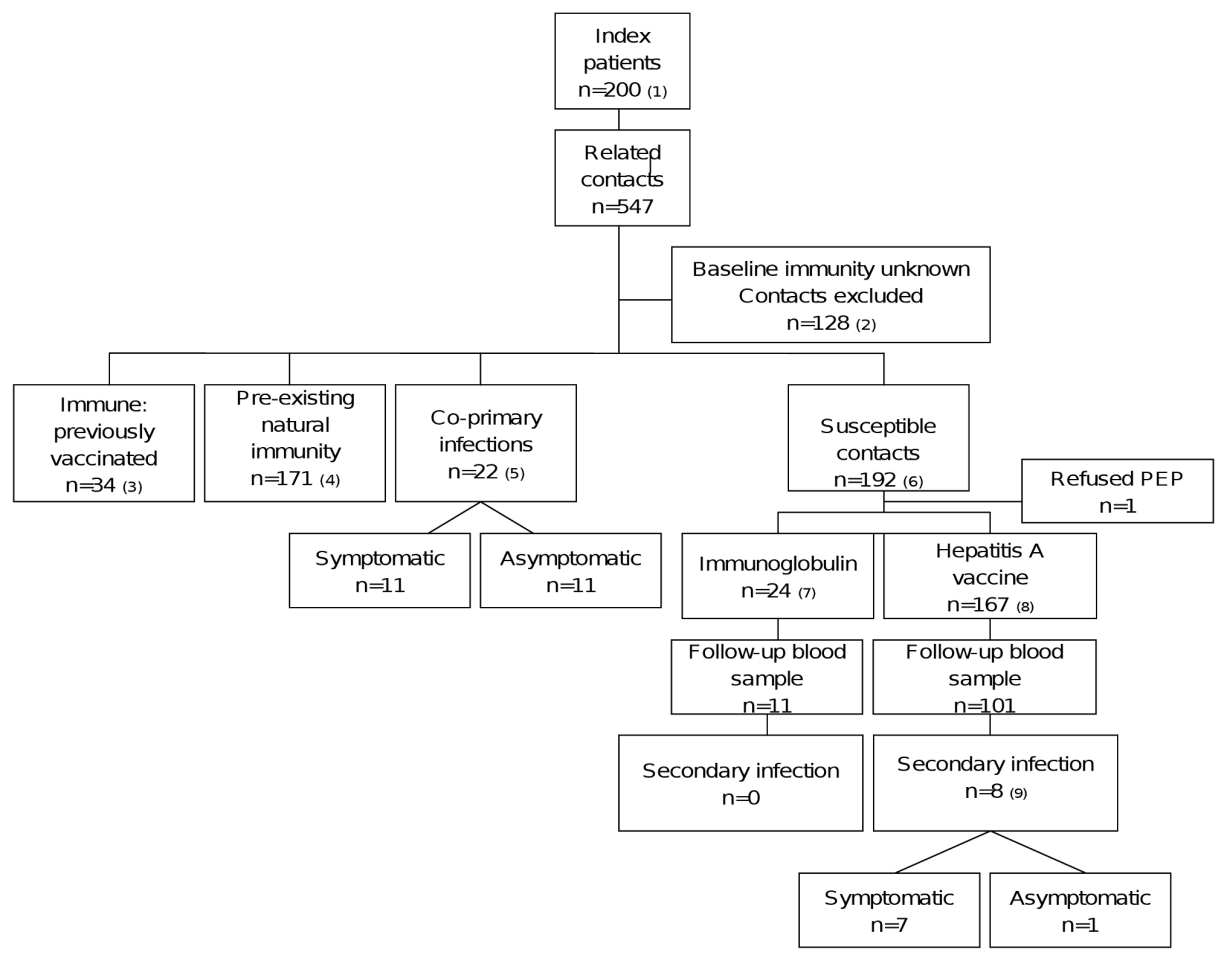
Algorithm of contacts identified, included, treatments assigned and outcomes. 1. Cases notified with clinical signs or symptoms of infection and elevated amino-transferase levels, with detectable hepatitis A-specific immunoglobulin M (IgM) antibodies in the serum (in the absence of hepatitis A vaccination in the last 12 months) or an epidemiological link to a confirmed case. 2. Baseline serological status was unknown for 128: 63 were not tested, 65 were tested ≥15 days post exposure. 3. Vaccinated twice (or once if within one year of exposure) with inactivated hepatitis A vaccine. 4. Asymptomatic and total anti-HAV positive within 14 days of exposure. If ≤10 years, also IgM negative. 5. All IgM positive ≤14 days post-exposure. 6. Total anti-HAV negative and without symptoms. 7 Immunoglobulin given as PEP according to guidelines. See Table 1. 8. Hepatitis A vaccine given as PEP according to guidelines. See Table 1. Total anti-HAV negative at baseline and anti-IgM positive with jaundice (+/- HAV RNA on PCR) at follow-up, or in the absence of jaundice, anti-IgM positive and HAV RNA detected by PCR in the same follow-up sample.

In the Netherlands, symptomatic HAV infection is notifiable and the Public Health Service (PHS) routinely traces contacts of cases, offering post-exposure prophylaxis (PEP) according to national guidelines. Since 2004, hepatitis A vaccine has been used routinely in post-exposure prophylaxis [4]. Until 2004, hepatitis A immunoglobulin (IG) was the only form of PEP recommended, and evidence dating largely from the 1940s to 1960s [5-8] and clinical experience since [9], supports its effectiveness. Widespread use of a human blood-derived immunoprophylactic has become controversial however [10,11], and in some countries its use is no longer recommended [12]. In the interim, a safe, immunogenic vaccine which provides long term protection has been developed [13,14] and evidence of its efficacy and effectiveness in preventing secondary infection is accumulating [15-17]. In a randomised controlled trial conducted in 2007 [18], which directly compared the vaccine and immunoglobulin, the vaccine was shown to be similarly effective in preventing laboratory confirmed, symptomatic secondary HAV infection in healthy persons 2-40 years old. While national advisory bodies are increasingly recommending HA vaccine as an alternative to IG in PEP [19-21], IG remains the PEP of choice in those considered at increased risk of severe disease: people with certain underlying conditions or those who are immunosuppressed, for example. Internationally however, guidelines diverge concerning what PEP should be used in healthy people over 40 years [4,12,21-24], where evidence of vaccine efficacy is lacking. In the Netherlands, based on a review of the evidence and expert consensus [25], contacts aged <30 years, or, those aged 30-50 years and vaccinated within 8 days post-exposure, are generally considered to be at low risk of severe infection and vaccination is recommended [4]. Otherwise human immunoglobulin is prescribed (details in Table 1). In this study we evaluate the routine use of hepatitis A vaccine as an alternative to immunoglobulin in prevention of secondary HAV infection in contacts considered at low risk of severe infection. 

**Table 1:** Risk categories of contacts for whom active vaccination or immunoglobulin is recommended in post-exposure prophylaxis, LCI Netherlands*.

**Risk categories**		**Recommended post-exposure prophylaxis**	
		Active immunisation^1^ (Hepatitis A vaccine)	Passive immunisation (IgG)
Age	<30 years, with interval <8 days^2^	X	-
	<30 years, with interval >8 days	X	-
	30-50 years, with interval <8 days	X	-
	30-50 years, with unknown interval, or interval longer than 8 days^3^	-	X
	>50 years^4^	-	X
Childcare centres, schools, institutions for intellectually disabled.	Childcare centres and institutions for mentally disabled: when one case occurs, group-members and contacts using the same toilet should be vaccinated^5^	X	-
	≥2 cases reported within a school within 6 weeks: class members and contacts using the same toilet should be vaccinated^5^	X	-
Special groups	Persons with increase risk of serious hepatitis A infection, irrespective of age and interval^6^	-	X
	Persons with an immunosuppressive condition^7^, irrespective of age and interval.	-	X

* This table is an adaptation of advice contained in the guideline for the control of hepatitis A infection in the Netherlands [4].

1 Active immunisation is not recommended for children under 1 year.

2 The interval for a family contact is the period between the date of onset of symptoms in the index and immunisation; for other contacts, it is the interval between first contact with the index case and immunisation.

3 Passive immunisation longer than 28 days post-exposure is probably no longer effective.

4 If exposure is ongoing, active immunisation can be given simultaneously.

5 This recommendation extends to class teachers and supervisors. If, during an epidemic active immunisation occurs too late or many cases are reported simultaneously, then active immunisation of parents and siblings should also be considered.

6 Active immunisation of parents and siblings should also be considered.

7 People with liver cirrhosis, hepatitis B & hepatitis C.

## Methodology

### Notified cases: Routine Surveillance Data

All index cases of hepatitis A notified to the Amsterdam PHS after the revised protocol was implemented in July 2004 until end-December 2012 were included in this evaluation. Criteria for notification of hepatitis A include clinical signs or symptoms of infection and elevated amino-transferase levels, with detectable hepatitis A-specific immunoglobulin M (IgM) antibodies in the serum (in the absence of hepatitis A vaccination in the last 12 months) or an epidemiological link to a confirmed case. The first case notified in a household or care-centre was considered the index case, and the date of onset of illness was the date on which the index case first had jaundice (conjunctival or dermal icterus, pale stools or dark urine). To characterise the index cases, their most likely source of infection was categorised as: [1] those infected in a highly HAV-endemic country during the previous 6 weeks (imported infections); (2) those infected in the Netherlands by a hepatitis A patient in their home, school or work environment (domestic infections); (3) those who were infected as a result of male homosexual activity during the previous 6 weeks; (4) those of unknown source.

### Contacts: co-infections and secondary cases

A contact of an index case was defined as any family or household member (or equivalent) who shared toilet facilities with the index case, any sexual partner of the case, or any person who took care of an HAV-infected child while the index case was infectious (7 days before through 7 after the onset of jaundice). Once identified, contacts were invited to the PHS and were asked about signs or symptoms of HAV infection. They were offered advice on hygiene precautions and PEP according to guidelines: a single dose of hepatitis A vaccination or 0.02mls of immunoglobulin per kilogram of body weight depending on their risk category (as summarised in Table 1) and the post-exposure interval (i.e. for a family contact, the number of days between the date of onset of jaundice in the index case and the date of immunisation, and for other contacts, the period between first contact with the index and immunisation). Baseline total anti-HAV antibody testing was conducted at the same visit. As the incubation period of HAV is typically 15 to 50 days, contacts for whom baseline tests were conducted ≥15 days post-exposure were excluded. Asymptomatic contacts >10 years of age who tested total anti-HAV positive ≤14 days post-exposure were considered to have pre-existing immunity and were not tested further. In symptomatic contacts (and all children under 10 years of age who may have an acute asymptomatic infection) a total anti-HAV positive result ≤14 days post-exposure was followed by an anti-IgM test. If anti-IgM positive and exposed to the same likely source as the index, these contacts were considered co-infections and were not tested further. All those that were total anti-HAV negative at baseline were considered susceptible to HAV infection and were invited for follow-up serological testing four to eight weeks later, earlier if symptomatic. All samples that were anti-HAV IgM positive at follow-up were tested for HAV virus RNA (PCR). Secondary cases were symptomatic i.e. those who had jaundice and who tested anti-HAV IgM positive at follow-up, or asymptomatic i.e. in the absence of jaundice, those who had HAV virus RNA detected in the same follow-up sample. 

### Ethics Statement

In the Netherlands, hepatitis A is a notifiable infectious disease under the Public Health Act of 2008 [26]. Under article 6c of this legislation, it is the duty of the PHS to conduct contact tracing (in translation from Dutch: “The mayoral college and municipal council members are responsible for the implementation of general infectious disease control including source finding and contact tracing in relation to notifications referred to in articles 21, 22, 25 and 26”). This is operationalised in the national infectious disease control guidelines [4]. As a matter of routine, all contacts who receive PEP are invited to return for a follow-up blood test 4-8 weeks later to ensure seroconversion. This is entirely voluntary and for this, contacts give verbal informed consent. As this study was an evaluation of routine practice, written informed consent, ethical approval or a formal waiver of the need of ethical approval was not required. 

### Laboratory methods

Total anti-HAV and anti-HAV-IgM was performed using the combined automated quantitative HAVT test in a VIDAS system (Biomerieux, Craponne, France). This is a 2 step ELISA competition method with fluorescent detection. Amplification of HAV RNA was performed on the same follow-up sample tested positive for IgM, by a reverse transcriptase reaction and PCR according to Tjon et al [27].

### Statistical analysis

Data are presented categorically in proportions. The mean and standard deviation, or the median and interquartile range (IQR) are used to summarize continuous data. Significant differences between proportions were assessed using a two-sided Fischer’s exact test, and between means using two-sided independent sample t-tests. In a sub-group analysis of those considered at low-risk of severe infection who were vaccinated <8 days post-exposure, the relative risk (RR) of secondary infection was estimated using Poisson regression with clustered robust standard errors to correct for correlation between individuals within households. If the Wald test p value was <0.05, the association was considered significant. Analyses were conducted with Intercooled Stata 11.1 (Stata Corp., College Station, Texas, USA). 

## Results (Figure 1)

### Notified Cases

From 1 July 2004 and 31 December 2012, 200 cases of acute HAV infection were notified, of whom 64% were male (n=129), and the median age was 29 (IQR:12-41). Signs of jaundice included conjunctival or dermal icterus (n=178/200, 89%), pale stools or dark urine (n=22/200, 11%). Overall, 48% (n=97) were imported infections, 17% (n=34) were homosexually acquired, 11% (n=23) were domestically acquired infections and 23% (n=47) were of unknown source. The majority (75%, n=170) were born in the Netherlands, a further 21 (10%) were of other western origin, 7 were from Turkey or North Africa (3%), and 23 (11%) were other non-western countries.

**Figure 2:**
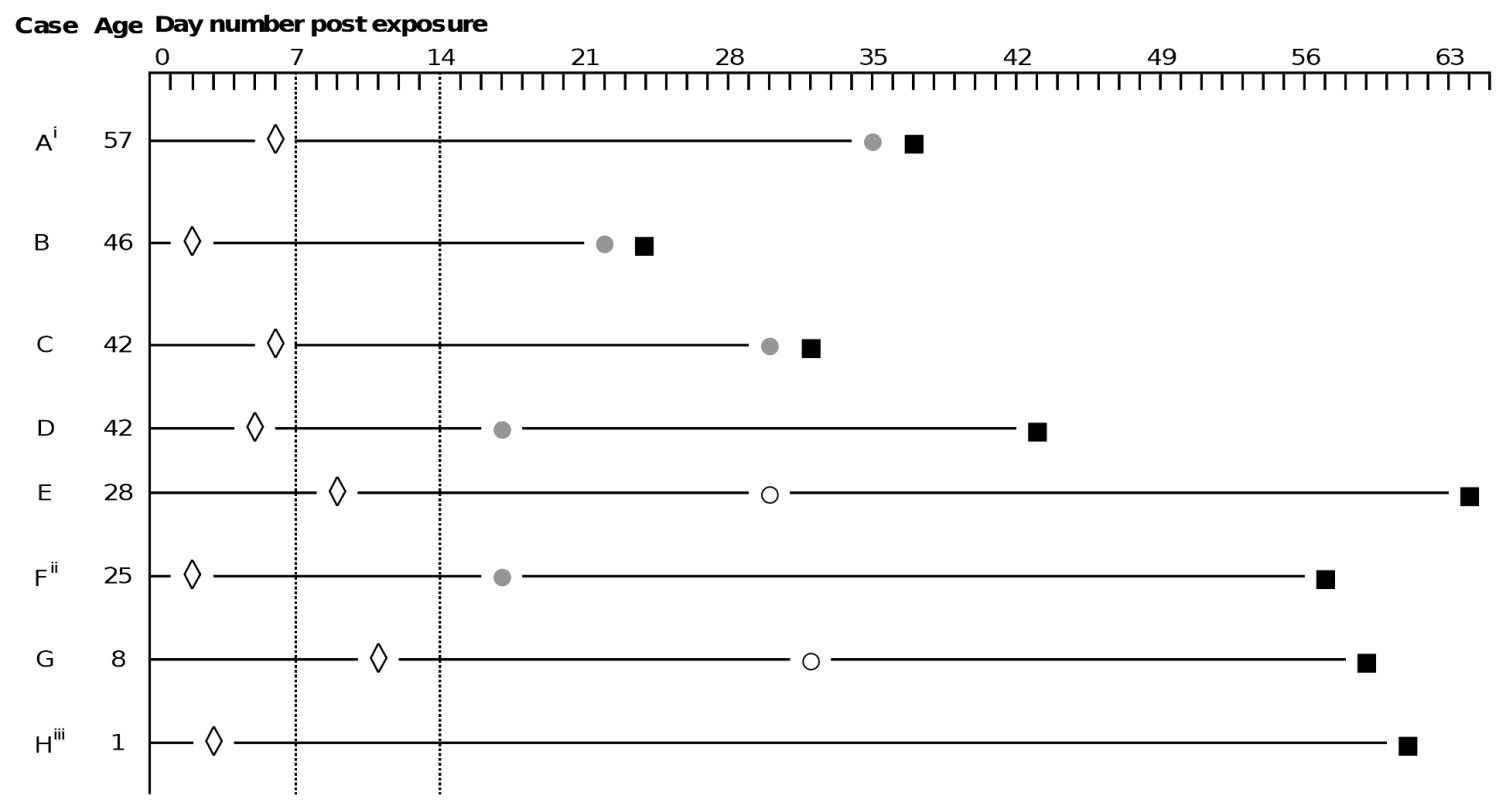
Timeline of exposure, vaccination, symptom onset and confirmation in secondary hepatitis A cases ◊ One dose hepatitis A vaccine administered. Total anti-HAV negative and asymptomatic. ● Jaundice (conjunctival icterus +/- dark urine, pale stools) ○ Non-specific symptoms: nausea, fatigue, loss of appetite, malaise ■ Date of confirmed infection: IgM positive & PCR positive. i Case was aged 57 and was not immunised according to protocol. ii This case was PCR negative, but IgM positive and symptoms and signs confirmed by general practitioner. iii Child aged 1 year who was asymptomatic throughout, but HAV RNA confirmed on PCR

### Contacts

In total, 547 contacts were identified (Figure 1) representing a median of 1 contact per case (IQR:0-4, range 0-24); 128 (23%) of contacts were excluded because blood samples were not taken, or blood was taken >14 days after symptom onset in the index. Characteristics of the remaining 419 contacts are in Table 2, of whom 205 (49%) were immune at presentation (34 had been fully vaccinated or vaccinated once, and 171 had pre-existing natural immunity). Similar to previous research [9], 84% (n=144/171) of those with pre-existing natural immunity were of Turkish, north African or other non-western descent (1^st^ or 2^nd^ generation) and 91% (n=159/171) were over 16 years. An additional 22 contacts were considered co-primary infections and were total anti-HAV and IgM positive ≤14 days post-exposure: 11 were contacts ranging in age from 2-45 years who complained of hepatitis A related symptoms, and 11 contacts were aged between 1 and 8 years and who were asymptomatic. 

**Table 2:** Characteristics of contacts whose baseline immune status was known (n=419).

Gender, n (%)		
	Female	218 (52)
	Male	201 (48)
Age (in years)		
	Mean (sd)	27.9 (19.1)
	Interquartile range	10-42
Visit to HAV endemic country during incubation period, n (%)		
	Yes	234 (56)
	No	178 (42)
	Unknown	7 (2)
Type of contact, n (%)		
	Sexual Partner^a^	41 (10)
	1st degree relative or equivalent household contact	294 (70)
	2nd degree relative^b^	58 (14)
	Other^c^	26 (6)
Duration, exposure to immunisation (days)		
	Mean (sd)	6.7 (3.3)
	Interquartile range Range	4-10
Hepatitis A status at baseline, n (%)		
	Previous natural immunity	171 (41)
	Vaccinated previously	34 (8)
	Co-infections	22 (5)
	Susceptible	192 (46)

a Of whom 7 were homosexual partnerships.

b Index was known to the contact and shared toilet facilities with or took care of the index, but was not a relative.

c Contacts were excluded if baseline blood was tested >14 days post-exposure

### Secondary cases

Overall, 192 contacts were susceptible at the time of presentation (Figure 1), of whom 167 (87%) were given hepatitis A vaccination within a median of 6 days (IQR:4-10) post-exposure, 24 (13%) got immunoglobulin within 10 days (IQR:4-14) and one refused PEP. Of 112 susceptible contacts who returned for follow-up testing, 18 (16%) were IgM HAV antibody positive, all of whom had received hepatitis A vaccine, and 8 of whom were considered secondary infections: 7 were symptomatic (5 made contact with the PHS complaining of yellowing of the eyes or skin, dark urine or pale stools among other symptoms; 2 contacts (aged 8 and 25) complained retrospectively of non-specific symptoms such as nausea, fatigue, loss of appetite when they returned for follow-up testing) and Hepatitis A virus RNA was confirmed by PCR in 6/7 of these cases (the PCR-negative case was a 25 year-old, diagnosed 17 days post-exposure by his general practitioner with conjunctival icterus, fever >38 celcius, and complaining of dark urine and pale stools. PCR testing was conducted 57 days post-exposure). In one additional IgM positive contact (a child aged 1 year who was asymptomatic throughout) HAV viral RNA was confirmed in the sample, bringing the total to 8 secondary cases (Figure 2). Of the remaining IgM positive but PCR negative cases (n=10), all were asymptomatic. All secondary cases were close family or household contacts (sexual partners, n=2, first degree relatives, n=6) and occurred in different family or household clusters. None were hospitalised. All had been vaccinated, including one 57 year old who was given hepatitis A vaccine in place of immunoglobulin 6 days post-exposure, for reasons that are unclear. Excluding this contact, 7.0% of those vaccinated per-protocol became secondary cases (n=7/100; 6/100 were symptomatic infections): 3% of those ≤15 years (2/58), 6% (2/32) aged 16-40 years, and 30% (3/10) >40 years. All three contacts over 40 developed clinically significant disease including jaundice. Among those followed-up who received immunoglobulin (n=11) the median age was 39 years (range 26-55). There were no secondary cases in this group.

### Risk factors associated with secondary infection

In a subgroup analysis of 72 healthy contacts aged <50 years who were vaccinated within 8 days post-exposure (Table 3), age was associated with an increased risk of secondary infection: compared to those aged ≤15 years the RR of secondary infection in those >40 years was 12.0 (95%CI:1.3-106.7). No association between gender, household size, ethnic background or the interval between exposure and vaccination, and secondary infection was shown in this group. 

**Table 3:** Factors associated with secondary infection in all contacts <50 years vaccinated within 8 days post exposure according to protocol (n=72).

		Vaccinated per-protocol <8 days post-exposure		Symptomatic secondary infection and/or PCR positive^a^			Univariable^c^	
							RR (95%CI)	p value^b^
		N		n	%			
**Total**		**72**		**5**	**6.9**			
Age group								
	<=15	40		1	2.5		1.0	
	16-40	22		1	4.5		1.8 (0.1-28.9)	
	41+	10		3	30.0		12.0 (1.3-106.7)	0.035
Gender								
	Female	36		4	11.1			
	Male	36		1	2.8		0.3 (0.0-2.2)	0.210
Household size^e^								
	2 persons	9		1	11.1		1.0	
	3-5 persons	29		3	10.3		0.9 (0.1-8.3)	
	6 or more persons	34		1	2.9		0.3 (0.0-4.1)	0.530
Ethnic background								
	Non-Western	38		2	5.3		1.0	
	Dutch/western	34		3	8.8		1.7 (0.3-10.2)	0.574
Interval between exposure & vaccination, mean days (standard deviation)		4.5 (1.7)		3.4 (2.6)			0.7^f^ (0.4-1.3)	0.223

a Of the 5 secondary infections included in this analysis, one was a child aged 1 who was asymptomatic, but PCR positive.

b. Wald test.

c All analyses are adjusted for clustering within households using clustered robust standard errors.

e 72 contacts were clustered in 44 households.

f The RR is the daily incremental risk.

### Exclusions and non-responders

Of those who were excluded (n=128), 63 did not have serological testing at baseline. The majority received PEP (n=44), all according to protocol and a mean of 5.5 days post-exposure (range 2-20 days). They were significantly younger than those who did have baseline serological testing (p=0.002) The remainder refused both serology and PEP (n=19). An additional 65 were excluded because PEP was administered and/or baseline blood taken ≥15 days post-exposure (median: 31.5 days, range 15-62): 33 (51%) had no history of symptoms and were immune at the time of testing, 24 (37%) were negative at first and follow-up testing, 8 were considered recent infections, testing total anti-HAV and anti-IgM positive at a median of 20 days post exposure (range 15-62 days). Of those who were susceptible and received PEP, but did not return for follow-up testing (n=80/191), there were no significant differences in age, gender or ethnic background nor in the exposure interval pre-PEP compared with those who did return. Of those excluded, no symptomatic cases were later reported to the PHS.

## Discussion

We evaluated the routine use of hepatitis A vaccine as an alternative to immunoglobulin in the prevention of secondary hepatitis A infection in contacts considered at low risk of severe infection over an 8-year period (2004-2012). In the Netherlands, all healthy contacts aged 1-30 years, and those aged 30-50 who are within 8 days of exposure to the index case, are given hepatitis A vaccine in preference to immunoglobulin. Of contacts vaccinated according to protocol, 6.0% seroconverted, developing a symptomatic laboratory confirmed, secondary infection. In the randomised controlled trial by Victor et al. [18], 4.4% of vaccinated contacts developed a symptomatic secondary infection. The trial included only contacts aged 2-40 years, and when the same age-group in our evaluation is compared, the proportion was similar (3.6%). Direct evidence of the efficacy of the vaccine in PEP in those over 40 is not available, so in the Netherlands, this recommendation was made based on a combination of epidemiological data, hospitalisation and case-fatality rates in the Netherlands, and vaccine immunogenicity data (seropositivity of 77% in persons aged 40-62 years post-vaccination in one study [28]). In our evaluation, 30% of contacts over 40 years vaccinated per-protocol developed symptomatic infection, and half of the 8 secondary infections were over 40 years, all of whom had been vaccinated. In a subgroup analysis of all healthy contacts vaccinated within 8 days of exposure, the risk of secondary infection in those over 40 years was 12 times that of contacts aged ≤15 years (95%CI:1.3-106.7). Internationally, guidelines vary regarding the upper age limit at which vaccination is offered in preference to immunoglobulin [21-24], and in some countries, vaccination is the only form of PEP offered [12]. Though in absolute terms, the number of secondary infections in this study is low, our findings suggest that a more conservative upper age limit for contact vaccination may be appropriate.

This real-world evaluation illustrates the complexities of post-exposure prophylaxis in a low-endemic country [29] where there are high levels of pre-existing immunity in some minority groups together with susceptible older adults at risk of severe infection. In the Netherlands, the incidence of acute hepatitis A infection has declined dramatically in recent years [30]. Although outcomes of this evaluation and that of 2004 are not directly comparable, the same definitions and timeframes were used throughout and the general picture in recent years is one of smaller households (median of 1 versus 3 family/ household contacts per index case), less pre-existing natural immunity among contacts (41% versus 54%), and a lower proportion of co-infections (11% versus 28%) among susceptible contacts. This picture, together with the overall decline in incidence, probably reflects reduced household exposure and transmission through improved household hygiene and reduced virus importation from countries where endemicity of HAV is decreasing as socioeconomic, sanitary and water supply conditions improve (Turkey and Morocco in particular). 

The proportion of IgM positive seroconversions was also reduced, 16%, versus 34% in the previous evaluation. In the latter (where only immunoglobulin was offered), only one-fifth of those who became anti-IgM positive were symptomatic, despite the 34% IgM-positive seroconversion rate. This was attributed to the attenuation of symptomatic infection by immunoglobulin. As vaccination can induce a transient IgM seropositivity [31]. we considered only those who were jaundiced and/or had detectable HAV RNA as true secondary infections. Our rate of secondary infection may therefore be an underestimate (though we think it unlikely that viral RNA would no longer be detectable given the short follow-up time). Irrespective, this would imply a relatively greater proportion of symptomatic infections among those who became IgM positive in our vaccinated population. Whether this reflects a real difference, a vaccine effect or a hygiene effect is uncertain. Ultimately, the rate of symptomatic, laboratory confirmed secondary HAV infection among susceptible contacts was similar in both evaluations: 5.3% (6/112 from 2004-2012) versus 6.4%(12/186 from 1996-2000).

There were a number of study limitations. Firstly, as in the previous evaluation, those who did not undergo baseline serological testing (and were thus excluded) were younger than those who participated. As children are more likely to be susceptible than adults, the proportion with pre-existing immunity may therefore be overestimated. Secondly, contacts ≥10 years old who were asymptomatic and total anti-HAV positive at baseline were assumed to be immune and were not screened for IgM antibody. This could have resulted in the misclassification of contacts who in fact were asymptomatically co-infected, though the majority of older children and young adults develop symptoms. Thirdly, symptomatic contacts may have been more inclined to return for follow-up testing, leading to an overestimate of the secondary infection rate, but in any case, the proportion of seroconversions was small. Finally, we did not find any association between gender, ethnic background or household size and secondary infection, but as the absolute number of secondary infections in this study was small, the study may have been underpowered. It is possible that factors other than age (level of education for example) contributed to secondary HAV infection in those over 40 years who were vaccinated within 8 days of exposure.

In conclusion, timely administration (≤14 days post-exposure) of HAV vaccine in routine post-exposure prophylaxis was feasible and the incidence of laboratory confirmed, symptomatic secondary infection in contacts of hepatitis A cases aged under 40 years was low. Although larger studies are required, it may be prudent to limit routine use of vaccination in those over 40 years of age, irrespective of the duration post-exposure. Retaining the use of immunoglobulin as PEP of choice in older people and those at risk of severe illness seems appropriate. 
